# Interactions of Tomato Chlorosis Virus p27 Protein with Tomato Catalase Are Involved in Viral Infection

**DOI:** 10.3390/v15040990

**Published:** 2023-04-18

**Authors:** Xiaohui Sun, Lianyi Zang, Xiaoying Liu, Shanshan Jiang, Xianping Zhang, Dan Zhao, Kaijie Shang, Tao Zhou, Changxiang Zhu, Xiaoping Zhu

**Affiliations:** 1 Shandong Province Key Laboratory of Plant Virology, Institute of Plant Protection, Shandong Academy of Agricultural Sciences, Jinan 250100, China; 2 College of Plant Protection, Collaborative Innovation Center of Fruit & Vegetable Quality and Efficient Production, Shandong Agricultural University, Taian 271018, China; 3 State Key Laboratory of Agrobiotechnology, Department of Plant Pathology, China Agricultural University, Beijing 100193, China; 4 State Key Laboratory of Crop Biology, College of Life Sciences, Shandong Agricultural University, Taian 271018, China

**Keywords:** tomato chlorosis virus, p27, catalase, virus host proteins

## Abstract

Tomato chlorosis virus (ToCV) severely threatens tomato production worldwide. P27 is known to be involved in virion assembly, but its other roles in ToCV infection are unclear. In this study, we found that removal of p27 reduced systemic infection, while ectopic expression of p27 promoted systemic infection of potato virus X in *Nicotiana benthamiana*. We determined that *Solanum lycopersicum* catalases (SlCAT) can interact with p27 in vitro and in vivo and that amino acids 73 to 77 of the N-terminus of SlCAT represent the critical region for their interaction. p27 is distributed in the cytoplasm and nucleus, and its coexpression with SlCAT1 or SlCAT2 changes its distribution in the nucleus. Furthermore, we found that silencing of *SlCAT1* and *SlCAT2* can promote ToCV infection. In conclusion, p27 can promote viral infection by binding directly to inhibit anti-ToCV processes mediated by SlCAT1 or SlCAT2.

## 1. Introduction

The tomato chlorosis virus (ToCV; genus *Crinivirus*, Closteroviridae family) is one of the major tomato (Solanum lycopersicum) viruses. It is particularly harmful to greenhouse tomatoes and seriously restricts the development of the tomato industry [1]. ToCV was first discovered in tomato plants in greenhouses in central Florida in the mid-1990s and then spread throughout the world by MEAM1 and MED biotypes of *Bemisia tabaci,* and the virus is transmitted by whiteflies in a semi-persistent manner [2,3]. ToCV can infect plant species from the families Solanaceae, Cucurbitaceae, Leguminosae, Apocynaceae, and Asteraceae [3]. The severe symptoms induced by this virus include interveinal chlorosis, chlorosis, thickened and brittle leaves, and weak plant growth [4,5]. The ToCV genome contains two single-stranded positive sense RNA molecules, including 8594 bp for RNA1 and 8242 bp for RNA2, encapsulated in filamentous flexuous virions [3]. The ToCV genome encodes a dozen proteins, but their functions still need to be well documented.

The p27 protein encoded by ToCV RNA2 has been reported to be involved in virion assembly, but it is unknown whether it is involved in other functions associated with ToCV infection [6]. Recently, homologs of p27, including p26 of lettuce infectious yellows virus (LIYV, genus *Crinivirus*) and the p33 protein of citrus tristeza virus (CTV, genus *Closterovirus*, Closteroviridae family), have been found to play essential roles in viral intercellular movement [7,8,9]. Furthermore, the yeast cotransformation method has verified the self-interaction of LIYV p26 and ToCV p27 [10].

Viral proteins interact with multiple host factors during pathogenesis, but further research is needed to determine how plant viruses induce specific disease symptoms [11]. Hydrogen peroxide (H_2_O_2_), one of the reactive oxygen species, plays a vital role in disease resistance [12]. Catalase breaks down H_2_O_2_ and acts as a cellular receiver for H_2_O_2_ in almost all organisms. In *Arabidopsis thaliana*, the family of catalase genes consists of CAT1, CAT2, and CAT3. Several recent studies have shown that viral proteins promote viral infection through different mechanisms by interacting with catalase. Triple gene block protein 1 (TGBp1) of the pepino mosaic virus interacts with and increases the activity of SlCAT1, improving virus accumulation [13]. The interaction between the 2b protein of cucumber mosaic virus and CAT3 induces a specific type of necrosis associated with proteasome activity [14]. With infection by the chilli veinal mottle virus (ChiVMV) in *Nicotiana tabacum*, CATs are activated. Then, HCPro interacts with CAT1 to inhibit CAT activity, resulting in the generation of H_2_O_2_, which aids in virus infection. Finally, the ROS burst induces systemic cell death in infected plants [15]. Maize CATs interact with maize chlorotic mottle virus (MCMV) p31 in yeast. The interaction between ZmCAT1 and P31 promoted viral accumulation. Mechanically, P31 attenuates the expression of salicylic acid (SA)-responsive pathogenesis-related genes (PR) by inhibiting catalase activity to allow for efficient viral infection [16].

This study demonstrated that p27 promotes systemic systemic ToCV infection in *N. benthamiana*. SlCAT1 and SlCAT2 could interact with ToCV p27 in vitro and in vivo, identifying key domains of ToCV p27 interaction with CATs. Silencing CATs promote RNA accumulation levels in ToCV. The severity of plant symptoms was positively correlated with ToCV accumulation, suggesting that the interaction between ToCV p27 and CAT may play an essential role in virus infection and pathogenicity.

## 2. Materials and Methods

### 2.1. Plant Growth Condition and Virus Inoculation

*N. benthamiana* plants were grown under a 16 h light/8 h dark regime at 25 °C. These plants were used for agroinfiltration in the four-leaf stage. *N. benthamiana* was inoculated with an infectious ToCV clone.

### 2.2. Construct Preparation

All primers used for plasmid construction are shown in Table S1. P27 was obtained from the infecting clone pCass4-Rz-ToCV-RNA2 by PCR amplification using Phusion DNA polymerase. The 150 bp CE control element of the ToCV p27 mutant was amplified by Phusion DNA polymerase from the infectious clone pCass4-Rz-ToCV-RNA2 by PCR. Primers were used to amplify PVX-Flag-p27-F/PVX-Flag-p27-R and construct an overexpression vector to overexpress p27. BD-p27-F/BD-p27-R, AD-SlCAT1-F/AD-SlCAT1-R, and AD-SlCAT2-F/AD-SlCAT2-R were used to verify the interaction of p27 with SlCAT1 and SlCAT2 in vitro. In vivo, YFP-N-p27-F/YFP-N-p27-R, YFP-C-SlCAT2-F/YFP-C-SlCAT2-R, and YFP-C-SlCAT1-F/YFP-C-SlCAT1-R were used to verify the interaction of p27 with SlCAT1 and SlCAT2. The 2-PROKII-p27-F/2-PROKII-p27-R, pGD-SLCAT1-Flag-F pGD-SLCAT1-Flag-R, and pGD-SlCAT2-Flag-F/pGD-SlCAT2-Flag-R primers were used for Co-IP verification interaction.

### 2.3. The Tomato cDNA Library Screening

A tomato cDNA library cloned into the pGADT7 vector (Shanghai OE Biotech Co., Ltd., Shanghai, China) was used in protein–protein interaction screens. The ToCV-p27 gene was amplified directly from the ToCV-BJ isolate using the primer pair BD-p27-F/BD-p27-R (Table S1). The recombinant plasmid pGBKT7-p27 was cloned into the PGBKT7 vector (Shanghai OE Biotech Co., Ltd., Shanghai, China) by double digestion. PGBKT7-p27 was transferred to the Y2H Gold strain, and then it was coated on a SD/-Trp medium plate and cultured in a 30 °C incubator for observation under 5 d. Single colonies were selected and added to 50 mL of SD/-Trp medium for shock culture for 20 h until the OD600 were 0.8 and then fused with 1 mL of the tomato library for 24 h. Microscopical observation observed the formation of zygotes in the shape of a clover, and the resuspended cells were centrifuged at low speed and added to YPDA. The bacterial solution (300 μL) was diluted to 3 mL with 0.9% NaCl containing Kan (50 μg/mL) and spread in SD/-Trp-Leu + X-α-Gal + 125 ng/mL of AbA medium plates. These plates were coated and cultured upside down in a 30 °C incubator for approximately five days to observe colony growth in the solid plates. Then, blue-white spot screening was performed on the SD/-Ade-His-Leu-Trp + X-α-Gal + 125 ng/mL AbA plate.

### 2.4. Bimolecular Fluorescent Complimentary Assay (BiFC)

In planta, confirmation of the ToCV-p27-YN + SLCAT1-YC, ToCV-p27-YN + SLCAT2-YC, ToCV-p27-YN + YC, YN + SLCAT1-YC, and YN + SLCAT2-YC interactions in *N. benthamiana* was performed using a BiFC assay. Subsequently, the constructs were transformed into GV3101. Further agroinfiltration was carried out by resuspending A. tumefaciens cells in MES-MgCl_2_ induction buffer (10 mM MES, 10 mM MgCl_2_, pH 5.6, 200 µM acetosyringone) at a final OD 600 nm of 0.5/0.5 for target proteins and 0.3 for p19 suppressor using a needleless syringe. The fluorescence of YFP in the leaves was observed by confocal laser scanning.

### 2.5. Co-Immunoprecipitation Assay (Co-IP)

IP buffer (50 mM Tris-HCl pH 7.5, 150 mM NaCl, 1 mM EDTA, 1% Triton X-100, 10% glycerol, 20 mL PMSF protease inhibitor) was used to extract total plant DNA and then centrifuged at 16,000× *g* 3 times at 4 °C for 10 min. It was vortexed for 1 min, and 25 µL beads were suspended in a 1.5 mL centrifuge tube. A binding buffer (200 µL) was added, and the solution was suctioned and mixed. Centrifuge tubes were placed on a magnetic separator for magnetic separation. The binding buffer was used to dilute the primary antibody. The working solution was prepared with a final concentration of 50 µg/mL and placed on ice for standby. After magnetic separation, 200 µL of antibody working solution and protein sample were added after washing with buffer. Then, it was absorbed, mixed, and placed on a rotating mixing instrument to turn the centrifugal tube for 1 h at room temperature; The solution was washed 5 times with IP buffer. Flag or GFP antibodies wereused for Western blot detection.

### 2.6. Confocal Microscopy

Subcellular location and BiFC were observed using a confocal Zeiss LSM880 microscope. After agrobacterium tumefaciens had infiltrated the leaves for 72 h, the epidermal cells of the *N. benthamiana* leaf were torn and made into temporary slides, which were placed on the loading platform for the confocal microscope. The corresponding laser length was adjusted, and the multitrack mode was used for multicolour fluorescence scanning shooting. The photos were processed with ZEN Blue Version 2.1.

### 2.7. RT-qPCR

Total RNA was extracted using the RNAiso Plus reagent from *N. benthamiana* leaves following the manufacturer’s protocol (TaKaRa, Tokyo, Japan). Reverse transcription was performed using EasyScript reverse transcriptase (TransGen Biotech, Beijing, China). Samples were used for the quantitative real-time detection of gene expression levels by RT-qPCR. Three independent organisms were evaluated in each experiment to give three technical replicates. EF-1α was used as an internal reference for gene expression analysis. The 2^−ΔΔCT^ method was used to calculate relative expression levels [17]. All primers used for RT-qPCR analysis are shown in Table S1.

### 2.8. Protein Extraction and Western Blot

The leaves were weighed and placed in 7.5% (*v*/*v*) mercaptoethanol, containing 75 mM Tris-HCL (pH6.8), 8 M urea, 4.5% (*g*/*v*) SDS, and 4.5% (*g*/*v*) SDS, while keeping the 1 g/5.5 L ratio unchanged in all samples. The protein *N. benthamiana* from the pooled buffer was extracted, and the suspension was incubated at 90 °C for 15 min. The suspension was centrifuged at 13,000× *g* for 1 min with 100 °C water bath samples and separated on 15% SDS-PAGE. One gel was stained with colloidal coomassie blue staining, and the other gel used a nitrocellulose membrane for 30 min at 200 mA using a semi-dry transfer device. The membrane was sealed with 3% skim milk in a PBS solution, supplemented with specific antibodies for 2 h at room temperature and incubated overnight at 4 °C.

## 3. Results

### 3.1. p27 Is Involved in ToCV Systemic Infection in N. benthamiana Plants

**Figure 1:**
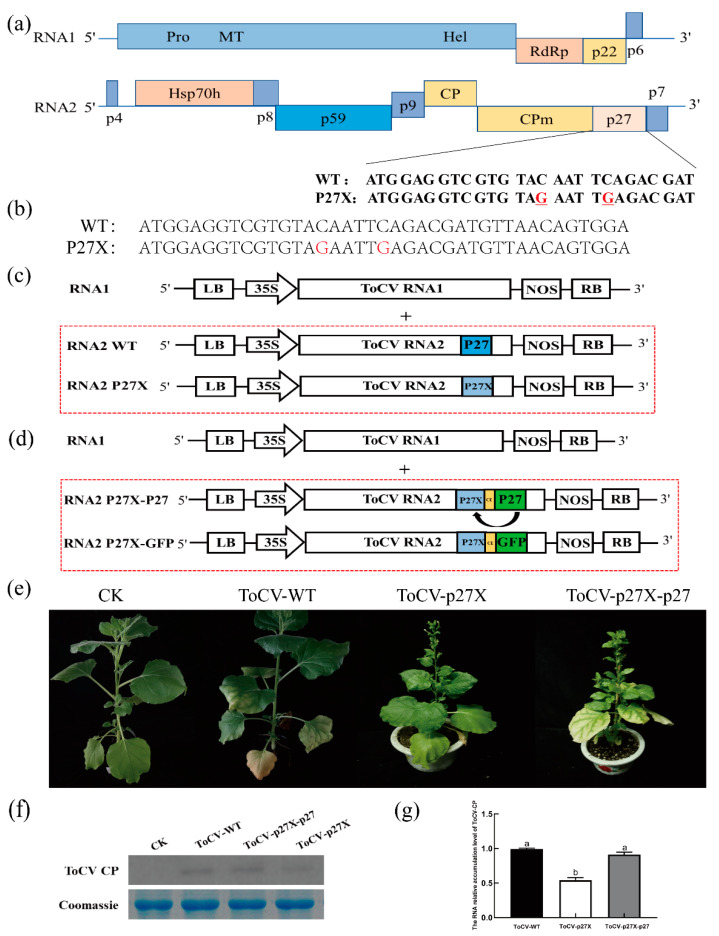
P27 promotes ToCV systemic infection in *N. benthamiana* plants. (**a**) Schematic diagram of the structure of the ToCV p27 base mutation. (**b**) Diagram of results of the sequencing of the p27 termination. (**c**) Schematic diagram of the genomic structure of ToCV-WT and ToCV-p27X. (**d**) Schematic map of the genomic structure of *N. benthamiana* infected with the ToCV-p27X system. (**e**) Four weeks after inoculation, WT, mutant, and anaplerosis ToCV caused symptoms in *N. benthamiana*. (**f**) Four weeks after ToCV immunosuppression, the expression of the ToCV CP protein in the leaves of the *N. benthamiana* system was detected by Western blot analysis. (**g**) Four weeks after inoculation, the accumulation of ToCV CP in *N. benthamiana* was detected by qRT-PCR.

To study the role of p27 in ToCV infection, the 15th and 20th bases of the p27 coding region were mutated to introduce two stop codons, TAG and TGA, leading to prestop translation of p27 in the ToCV infectious clone ToCV (Figure 1a). The ToCV p27 mutant was named ToCV-p27X (Figure 1b). Furthermore, to produce complementary expression of p27 during ToCV infection, a 150-bp control element (CE) and an open reading frame (ORF) were inserted after the ORF constructs of p27X, obtaining ToCV-p27X-p27 (Figure 1c, d). The wild type (ToCV-WT), the mutant type (ToCV-p27X), and the complementary type (ToCV-p27X-p27) were inoculated separately in *N. benthamiana*. Four weeks after inoculation, yellowing and chlorosis symptoms occurred in the interveinal region of the lower leaves of all inoculated *N. benthamiana*. Compared to ToCV-WT plants, the symptoms of the ToCV-p27X mutant plants were weakened, while the symptoms of the ToCV-p27X-p27 anaplerosis plants remained unchanged (Figure 1e). Western blot showed that the ToCV CP protein was expressed in systemic leaves of *N. benthamiana* plants inoculated with ToCV-WT, ToCV-p27X, and ToCV-p27X-p27 (Figure 1f). The qRT-PCR results showed that the accumulation of CP in the ToCV-p27X mutant was 55.5% of that of ToCV-WT. The accumulation of CP in ToCV-p27X-p27 with p27 was 90% of that in ToCV-WT (Figure 1g). These results suggest that ToCV-p27X can restore its effect on ToCV infection by complementing p27.

### 3.2. Ectopic Expression of p27 Promotes PVX Systemic Infection in N. benthamiana Plants

**Figure 2:**
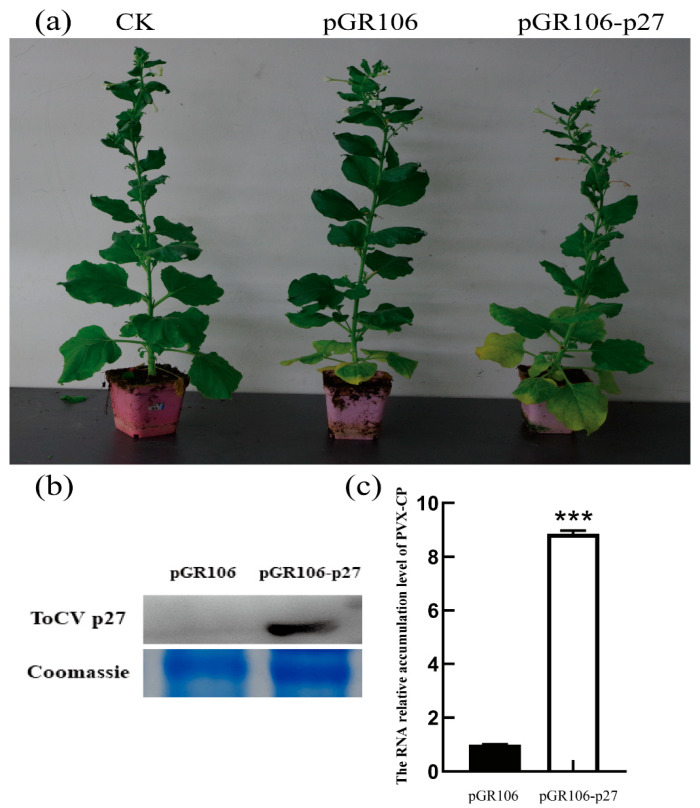
Expression of the ToCV p27 protein in *N. benthamiana* after inoculation with pGR106-p27. (**a**) On the 14th day after inoculation, the symptoms of *N. benthamiana* were inoculated with pGR106 and the pGR106-p27 virus. (**b**) Expression of the ToCV p27 protein in *N. benthamiana* 14 days after inoculation with pGR106-p27. (**c**) The accumulation of PVX *CP* in *N. benthamiana* was determined by qRT-PCR at 14 dpi. Error bars indicate standard deviation and statistical significance was calculated with Student’s *t*-test (*** *p* < 0.001).

*N. benthamiana* was inoculated with PVX (pGR106) or pGR106-p27. At 14 days after inoculation, *N. benthamiana,* infected with pGR106-p27, showed more severe mosaic symptoms than *N. benthamiana* infected with pGR106 (Figure 2a). The total protein content was extracted, and the Flag antibody was used to detect the p27 protein by Western blot analysis (Figure 2b). The qRT-PCR results show that the expression of PVX *CP* in pGR106-p27 was 8.75 times that of pGR106 (Figure 2c). Plants inoculated with pGR106-p27 showed more severe symptoms than those inoculated with pGR106, suggesting that p27 could promote PVX infection of *N. benthamiana*.

### 3.3. ToCV p27 Interacts with Tomato SlCAT1 and SlCAT2

**Figure 3:**
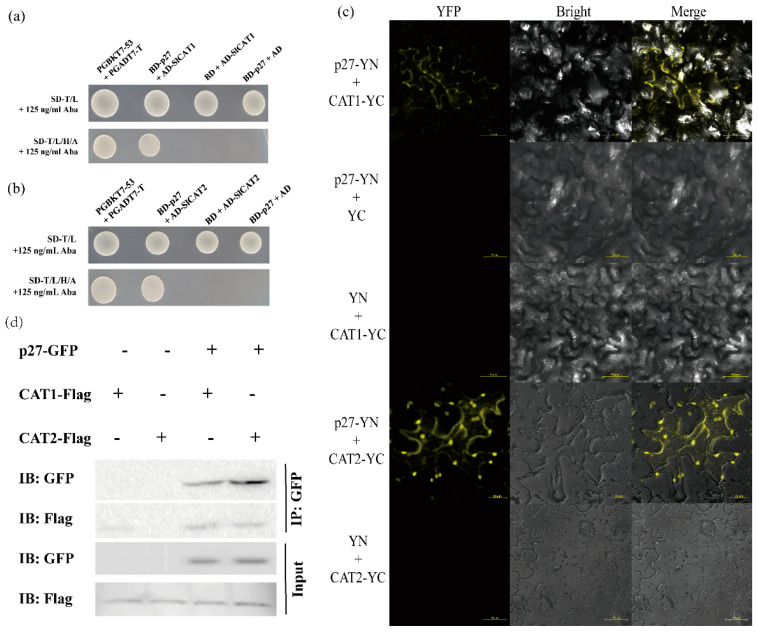
ToCV p27 interacts with itself and with SlCAT1 and SlCAT2. (**a**) Y2H verified the interaction of ToCV p27 with SlCAT1. (**b**) Y2H verified the interaction of ToCV p27 with SlCAT2. (**c**) BiFC verification shows that ToCV p27 interacts with SlCAT1 and SlCAT2. (**d**) Co-IP verified that ToCV p27 interacts with SlCAT1 and SlCAT2.

The interaction between virus protein and host protein can affect disease development. YTHS identified the *Solanum lycopersicum* protein that interacts with ToCV-p27. Sequence analysis indicated that the candidate protein *Solanum lycopersicum* catalase 1 (SlCAT1) could interact with p27. The interaction between p27 and SlCAT1 was verified by yeast cotransformation (Figure 3a). Solanum lycopersicum isoform catalase 2 (SlCAT2), homologous to SlCAT1, was found in tomato, and the consistency of the amino acid sequence between the two was 75.81%. The co-transformation of yeast verified the interaction between p27 and SlCAT2 (Figure 3b).

Furthermore, we verify the interaction between p27 and SlCAT1 or SlCAT2 by BiFC. The leaf cells of *N. benthamiana* that co-infiltrated ToCV-p27-YN + SLCAT1-YC and ToCV-p27-YN + SLCAT2-YC were detected by laser confocal microscopy. Strong yellow fluorescence was observed, and the yellow fluorescence was distributed in the cytoplasm. No fluorescence was observed in tissues of *N. benthamiana* infiltrated with negative controls ToCV-p27-YN + YC, YN + SLCAT1-YC, and YN + SLCAT2-YC (Figure 3c). These results indicate that p27 could interact with SlCAT1 and SlCAT2 in plants.

To further confirm the interaction in vivo, we conducted a Co-IP experiment. Combinations of ProkII-p27 and SLCAT1-Flag and ProkII-p27 and SLCAT2-Flag infiltrated the leaves of *N. benthamiana*. Three days later, Western blot analysis showed that all proteins could be detected (Figure 3d). The results of the CoIP experiment showed that p27 could interact with p27, SlCAT1, and SlCAT2.

### 3.4. The SlCAT1 N-Terminal Is Critical for The Interaction between SlCAT1 and p27

**Figure 4:**
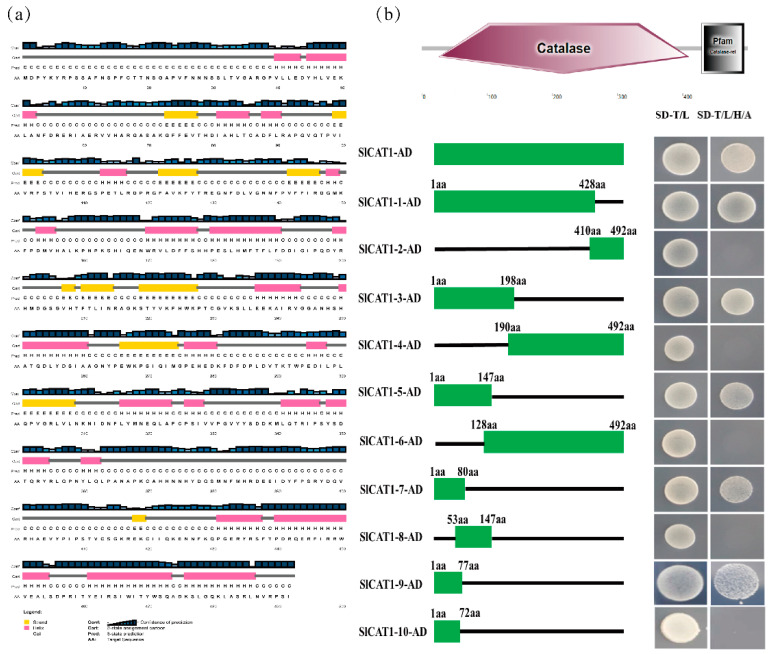
Verification of the functional region of the yeast cotransformation and the p27 interaction. (**a**) Prediction of the secondary structure of SlCAT1. (**b**) Prediction of the SlCAT1 domain, and Y2H was used to verify the interaction between p27 and the SlCAT1 deletion mutants.

To map the functional region of interaction between p27 and SlCAT1, a series of SlCAT1 deletion mutants was constructed, according to the results of the secondary structure prediction of SlCAT1 (Figure 4a) and the structural domain prediction (Figure 4b). pGADT7-SlCAT1Δ1 (1–428aa), pGADT7-SlCAT1Δ2 (410–428aa), pGADT7-SlCAT1Δ3 (1–198aa), pGADT7-SlCAT1Δ4 (190–492aa), pGADT7-SlCAT1Δ5 (1–147aa), pGADT7-SlCAT1Δ6 (128–492aa), pGADT7-SlCAT1Δ7 (1–88aa), pGADT7-SlCAT1Δ8 (53–147aa), pGADT7-SlCAT1Δ9 (1–77aa), and pGADT7-SlCAT1Δ10 (1–72aa) were used to determine the region in which SlCAT1 participates in the interaction. The ability of these mutants to interact with p27 was verified by cotransformation of yeast. The results showed that 73–77aa of the N-terminal is critical for self-interaction with SlCAT1 (Figure 4b).

### 3.5. SlCAT1 or SlCAT2 Changes the Location of p27

To investigate the subcelluar distribution of SlCAT1, SlCAT2, and p27, we separately expressed ProkII-p27, dsRed-SlCAT1, and dsRed-SlCAT2. p27 was found to be located in the cytoplasm and nucleus of epidermal cells of *N. benthamiana*. SlCAT1 and SlCAT2 were found to be localised in the cytoplasm and were shown to form many aggregates.

**Figure 5:**
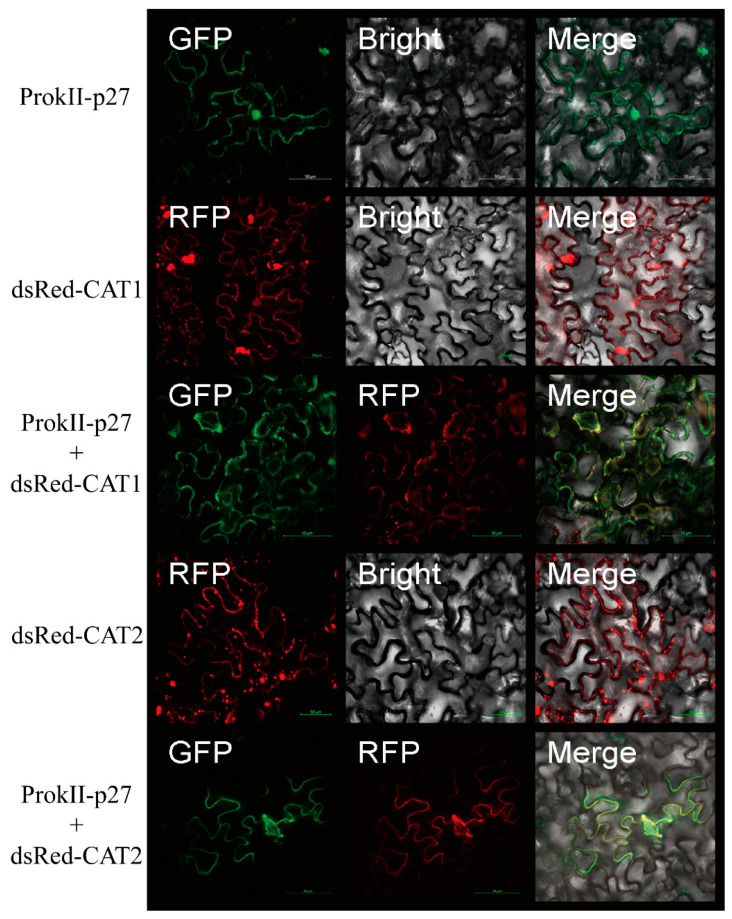
The expression of SlCAT1 and SlCAT2 changes the localisation of p27.

Furthermore, we investigate whether coexpression can change the subcellular distribution of p27 and SlCAT1 or SlCAT2. The results showed that p27 co-locates with SlCAT1 or SlCAT2 in aggregated particles in the cytoplasm (Figure 5). These results suggest that SlCAT1 or SlCAT2 could change the location of p27.

### 3.6. Silencing CAT Promotes ToCV Infection in N. benthamiana

**Figure 6:**
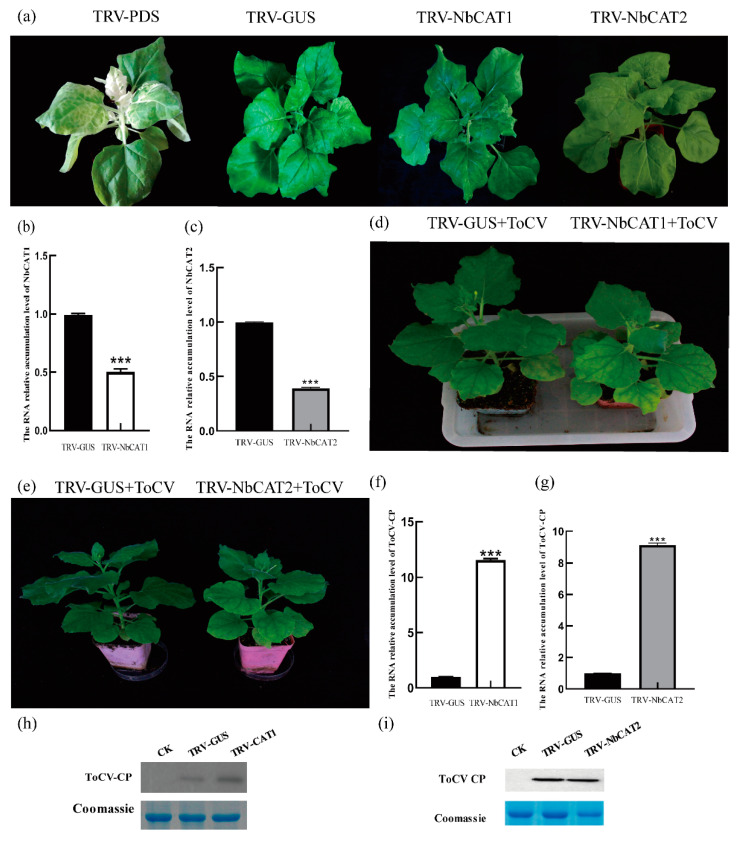
TRV-mediated silencing of the *NbCAT1* gene promotes ToCV infection of host plants. (**a**) Phenotype of *N. benthamiana* with TRV-mediated silencing of the *NbCAT1* and *NbCAT2* gene. (**b**) Detection of the silencing efficiency of the *NbCAT1* gene in *N. benthamiana* by qRT-PCR at 10 dpi. (**c**) Detection of the silencing efficiency of the *NbCAT2* gene in *N. benthamiana* by qRT-PCR at 10 dpi. (**d**) The ToCV symptom in *N. benthamiana* silent in *NbCAT1.* (**e**) The ToCV symptom in *N. benthamiana* silent of *NbCAT2.* (**f**) The relative accumulation of ToCV *CP* in *N. benthamiana* without *NbCAT1* by qRT-PCR. (**g**) The relative accumulation of ToCV *CP* in *N. benthamiana* silent of *NbCAT2* by qRT-PCR. (**h**) Western blot detection showing the expression of the ToCV CP protein in the upper leaves of *N. benthamiana* silent of *NbCAT1* at 20 dpi. (**i**) Western blot detection showing the expression of the ToCV CP protein in the upper leaves of *N. benthamiana* silent of *NbCAT1* at 20 dpi. Error bars indicate standard deviation and statistical significance was calculated with Student’s *t*-test (*** *p* < 0.001).

In *N. benthamiana*, *NbCAT1* and *NbCAT2* coding sequences were selected, and the TRV vector was inserted to construct the recombinant vectors TRV2-NbCAT1 and TRV2-NbCAT2. Meanwhile, TRV2-PDS was used as a positive control, and TRV2-GUS was used as a negative control. The recombinant vector was transferred to agrobacterium tumefaciens. The leaves of *N. benthamiana* were infiltrated with TRV1 to form combinations of TRV-PDS, TRV-GUS, TRV-NbCAT1, and TRV-NbCAT2. Ten days after inoculation, the top leaves of *N. benthamiana* inoculated with TRV-PDS showed obvious albinism, whereas the top leaves of *N. benthamiana* inoculated with TRV-GUS showed no obvious albinism. The leaves of *N. benthamiana* inoculated with TRV-NbCAT1 and TRV-NbCAT2 showed obvious mottling of chlorosis at the top (Figure 6a). The results of quantitative real-time fluorescence PCR (qRT-PCR) showed that, on the 10th day after TRV-NbCAT1, the silencing efficiency of the NbCAT1 gene was 50.35% (Figure 6b), indicating that the constructed silencing vector could effectively silence the NbCAT1 gene. QRT-PCR showed that, on the 10th day after TRV-NbCAT2, the silencing efficiency of the *NbCAT2* gene was 61.5% (Figure 6c), indicating that the constructed silencing vector could effectively silence the NbCAT2 gene.

The *N. benthamiana* plants were further inoculated with ToCV. *NbCAT1*-silenced *N. benthamiana* plants showed more severe chlorosis after inoculation with ToCV than mock plants (Figure 6d). The *N. benthamiana* plants, with silenced *NbCAT2,* showed a similar phenomenon after inoculation with ToCV as those with silenced *NbCAT1* (Figure 6e). The results of qRT-PCR showed that the level of RNA accumulation of ToCV CP RNA accumulation in the systemic leaves of the plant was 12.45 times higher than the mock plants (Figure 6f). The qRT-PCR results showed that the level of ToCV CP RNA accumulation in the systemic leaves of the plant was 8.95 times higher than mock plants (Figure 6g). At 20 dpi, the results of the Western blot showed that the accumulation of CP in the upper leaves of the control plant was lower than in the silenced plants of *NbCAT1* and *NbCAT2* (Figure 6h,i). These results indicate that the silencing of NbCAT1 and NbCAT2 is beneficial for ToCV infection.

## 4. Discussion

This study demonstrated that p27 plays an essential role in ToCV infection. The deletion of p27 inhibited ToCV infection, and the infection ability of ToCV mutants was restored after p27 supplementation. CAT1 inhibited ToCV infection by interacting with p27.

To clarify the role of ToCV infection, we constructed p27 knockout mutants, and complementary p27 mutants were constructed to determine whether the expression of p27 of p27 could be restored. The results showed that the accumulation of CP in ToCV plants inoculated with mutant p27 was significantly down-regulated, and the accumulation of CP in ToCV plants inoculated with supplemented p27 returned to the level of wild types, indicating that the loss of p27 reduced the accumulation of ToCV and that p27 could promote ToCV infection in host plants. This effect can be recovered by supplementing the expression of p27. p27 overexpressed by the PVX vector and the results showed that p27 enhanced PVX infection in *Nicotiana benthamiana* after 14 dpi. Research shows that studying the function and action of p27 is valuable.

P27 plays an essential role in ToCV infection. Few studies have investigated the interaction between ToCV and the host protein. In this study, the p27 protein was used as a decoy protein, and self-activation and toxicity were detected. The results showed that the recombinant vector was not toxic to yeast cells and had a slight self-activation. An AbA concentration of 125 ng/mL can inhibit self-activation and detect proteins that interact with PGBKT7-p27. Forty-three host proteins were identified from the tomato cDNA library using the Y2H technique. These may involve multiple biological pathways, such as transcriptional regulation, elongation regulation, glycolysis, and hormone metabolism during ToCV infection. Studies on the homologous gene p26 in representative LIYV species of the same genus found that p26 plays an essential role in the viral cell-to-cell movement related to PLD. It does not participate in the replication of viruses; instead, it is involved in frequent infection of viruses. The function of LIYV p26 provides a direction for the study of ToCV p27. Studies on CTV-p33, a homologue of ToCV-p27, have revealed that the p33 protein is a multifunctional protein with some characteristics of viral motion proteins, which are necessary for the infection of citrus hosts. The absence of ORF p33 interferes with the virus transfer rate to remote uninoculated plant tissues, suggesting that p33 affects the systematic transmission efficiency of the CTV virus and is a movement-related protein [7].

Among the identified proteins that interact with p27, SlCAT1 was repeated 23 times in the sequencing results. SlCAT1 is a terminal oxidase. It is one of the critical enzymes in the biological defence system. The prominent role of CAT in plants is to remove H_2_O_2_ generated in photorespiration and mitochondrial electron transfer so that cells can avoid the toxicity of H_2_O_2_ [18]. CAT is significantly expressed in photosynthetic tissues, vascular tissues, seeds, and reproductive tissues [19]. The expression and activity of CAT are affected by environmental factors, such as drought and high salinity, and biological factors, such as pathogens. It is regulated by time and space, which are crucial elements for plant growth and development [20].

To demonstrate the importance of SlCAT1 for plant growth and development, the silencing of NbCAT1 was mediated by the TRV vector. The results showed that obvious symptoms appeared in *N. benthamiana* after silencing of NbCAT1, with prominent chlorotic mottling occurring in the upper leaves of *N. benthamiana*, which became more and more evident as time passed. The symptoms became more and more severe. NbCAT1 has been proven to be essential for the growth and development of *N. benthamiana*. Subsequently, ToCV was inoculated with *N. benthamiana* plants. The accumulation of virus particles increased significantly, proving that down-regulation of NbCAT1 expression in tobacco plants is beneficial for ToCV infection of host plants. The protein structure of SlCAT1 was then predicted and analysed. SlCAT1 is 1479 bp in length and contains 492 amino acids. There are no signal peptides or transmembrane domains, and SlCAT1 is a tetramer hemoglobin composed of four identical peptide chain subunits with a conserved activation centre and myoglobin binding site. The Y2H, BiFC, and Co-IP experiments verified the interaction between SlCAT1 and p27. The results showed that p27 could interact with SlCAT1 in vivo and in vitro.

There are different numbers of CAT family proteins in many plants, from as few as two to as many as ten. For example, there are three proteins in *N. benthamiana*, namely, NbCAT1, NbCAT2, and NbCAT3. CAT1 is expressed mainly in leaves and can remove hydrogen peroxide produced by photorespiration [21]. The expression of CAT2 is induced by ozone and pathogens and plays an essential role in plant stress resistance [22]. It mainly removes hydrogen peroxide produced by the oxidative metabolism of fatty acids. There are two CATs in tomato. Taking into account SlCAT2, the same family protein interacts with p27 in the same way as in SlCAT1. SlCAT2 has been partially studied. The consistency of the amino acids of SlCAT2 and SlCAT1 was found to be 75.81%. The results showed that p27 could interact with SlCAT2 in vitro and in vivo through yeast cotransformation, BiFC, and Co-IP experiments. At the same time, subcellular localisation and colocalisation of p27, SlCAT1, and SlCAT2 were carried out. p27 was found to be located in the cytoplasm and nucleus, while SlCAT1 and SlCAT2 were shown to be localised in the cytoplasm and formed many aggregation particles. During colocalisation, the presence of SlCAT1 and SlCAT2 changed p27 and caused colocalisation in the cytoplasm. These results indicate that SlCAT1, SlCAT2, and p27 can interact in vivo and that the interaction may occur in the cytoplasm. To further determine the functional region of the interaction between p27 and SlCAT1, a series of deletion mutants were constructed for SlCAT1, and the N-terminal of SlCAT1 was confirmed to be the central region involved in the interaction between p27 through a series of yeast cotransformation experiments.

P27 promotes cell-to-cell movement in ToCV. The functional regions of p27 interact with SlCAT1 by building deletion mutants. CAT has been shown to affect the salicylic acid pathway in other crops. Future studies will look at the effect of ToCV infection on salicylic acid and determine how salicylic acid responds to ToCV infection. This study has laid the foundation for the discovery of the protein function of p27 and the study of the interaction between ToCV and host plants.

## 5. Conclusions

In summary, we proven that p27 could promote virus infection. Y2H preliminarily verified the interaction between SlCAT1 and p27, and BiFC and Co-IP verified the interaction between SlCAT1 and p27. The interaction between p27 and SlCAT2, a protein of the same family as SlCAT1, was verified by Y2H, BiFC, and Co-IP. P27 is located in the cytoplasm and nucleus, and SlCAT1 and SlCAT2 are located in the cytoplasm and form many aggregates of small particles. The colocalization of SlCAT1 and SlCAT2 with p27 changed the distribution of p27 in the cell, causing the colocalization in the cytoplasm and forming some aggregated particles. SlCAT1 and SlCAT2 interact with p27 in the cytoplasm. By silencing the NbCAT1 and NbCAT2 gene of *N. benthamiana* plants, the importance of NbCAT1 and NbCAT2 in the growth and development of *N. benthamiana* has been proven, and the silencing of NbCAT1 and NbCAT2 is beneficial to ToCV infection in *N. benthamiana*.

## Data Availability

All data used in this study are already provided in the manuscript in its required section. There are no underlying data available.

## Supplementary Materials

The following supporting information can be downloaded at: https://www.mdpi.com/article/10.3390/v15040990/s1, Table S1: Primers used in this study.
