# Supplementary material for: Interactions of Tomato Chlorosis Virus p27 Protein with Tomato Catalase Are Involved in Viral Infection
Source: Viruses. 2023 Apr 18;15(4):990. doi: 10.3390/v15040990 (PMC10145342; doi:10.3390/v15040990)
Supplement: Supplementary file 1 [file viruses-15-00990-s001.zip › viruses-1941122-supplementary.pdf]

**Table S1 Primers used in this study**

| Primer | Primer sequence | Purpose |
|---|---|---|
| PVX-Flag-p27-F | CACCAGCTAGCATCGATATGGATTACAAGGATGACGACGATAAGGAGGTCGTGTACAATTCAGACG | Overexpression |
| PVX-Flag-p27-R | TTCATCGGCGGTCGACTCACTTATCGTCGTCATCCTTGTAATCAATATAATAATTTGAGACTCTT | |
| PVX-1457-F | TCACGCAGCAAAAGAAAGGC | RT-PCR detection |
| PVX-4667-R | CTCACCTTGTCAGGGACACC | |
| ToCV p27X-R5 | TCAAATATAATAATTTGAGACTCTTTCTATATTG | p27 point mutation |
| ToCV p27X-F6 | TTTTCAATATGATTTCCACTTATTTTACC | |
| TB1520-1-F | CGGTTGGGAATTTCATTTACCATGGGACGCGATTGGAA | p27 point mutation |
| TB1520-1-R | CGTCTCAATTCTACACGACCTCCATCGATCA | |
| TB1520-2-F | GGTCGTGTACAATTGAGACGATGTTAACAGTGGAGTT | p27 point mutation |
| TB1520-2-R | GTCACTTATCTTATCTTTCGCGAAGAACTTAATGACAGTGAAGACG | |
| HBJC-CE+p27-F | ATGGAGAACAGTGCTGTTGC | p27 point mutation |
| HBJC-CE+p27-R | TCAAATATAATAATTTGAGA | |
| ToCV p27X-F1 | TGGGAATTTCATTTACCATGGGACGCGATTGGAAAGTATGGA | p27 point mutation |
| ToCV p27X-R1 | AACCGGATCAAACTCCACTGTTAACATCGTCT | |
| ToCV p27X-F2 | CAGTGGAGTTTGATCCGGTTAAGATGTAAATACGACCG | p27 point mutation |
| ToCV p27X-R2 | CACTTATCTTATCTTTCGCGAAGAACTTAATGACAGTGAAGACGA | |
| ToCV p27X-CE-GFP-F3 | TCTCAAATTATTATATTTGAATGGAGAACAGTGCTGTTGCAA | p27 point mutation |
| ToCV p27X-CE-GFP-R3 | CCCTTGCTCACCATTCGATTAGGATTAGCCGGAATG | |
| ToCV p27X-CE-GFP-F4 | AATCGAATGGTGAGCAAGGGCGAGG | p27 point mutation |
| ToCV p27X-CE-GFP-R4 | AGTGGAAATCATATTGAAAATTACTTGTACAGCTCGTCCATGCC | |
| ToCV p27X-CE-p27-F3 | TCTCAAATTATTATATTTGAATGGAGAACAGTGCTGTTGCAA | p27 point mutation |
| ToCV p27X-CE-p27-R3-1 | CGACCTCCATTCGATTAGGATTAGCCGGAATG | |
| ToCV p27X-CE-p27-F4-1 | TCCTAATCGAATGGAGGTCGTGTACAATTCAGACG | p27 point mutation |
| ToCV p27X-CE-p27-R4-1 | AGTGGAAATCATATTGAAAATCAAATATAATAATTTGAGACTCTTTCTATATTG | |
| 1577-R | GCAACAGCACTGTTCTCCATTCAAATATAATAATTTGAGACTCTT | p27 point mutation |
| 108-F | CTCAAATTATTATATTTGATTTTCAATATGATTTCCACTTATTTTACC | |
| BD-p27-F | TCAGAGGAGGACCTGCATATGATGGAGGTCGTGTACAATTC | Y2H |
| BD-p27-R | CTAGTTATGCGGCCGCTGCAGTCAAATATAATAATTTGAGA | |
| AD-SlCAT1-F | GAGGCCAGTGAATTCATGGATCCCTCTAAGTATCG | Y2H |
| AD-SlCAT1-R | GAGCTCGATGGATCCTCACATTGTAGGCTTCACAG | |
| AD-SlCAT2-F | GAGGCCAGTGAATTCATGGATCCTTACAAGTACCGTC | Y2H |
| AD-SlCAT2-R | GAGCTCGATGGATCCTCATATGCTTGGTCTCACATTA | |

| | | |
|---|---|---|
| AD-SlCAT1(1-1284)-F | gtaccagattacgctcatatgATGGATCCATACAAGTATCG | Y2H |
| AD-SlCAT1(1-1284)-R | atgcccacccgggtggaattcAAAATTGTTCTCTTTCTGAATGAC | |
| AD-SlCAT1(1228-1476)-F | gtaccagattacgctcatatgTCACAATGTGCACTGGAAAACG | Y2H |
| AD-SlCAT1(1228-1476)-R | atgcccacccgggtggaattcTATGCTTGGTCTCACATTAAGCCTAG | |
| AD-SlCAT1(1-594)-F | gtaccagattacgctcatatgATGGATCCATACAAGTATCG | Y2H |
| AD-SlCAT1(1-594)-R | atgcccacccgggtggaattcATCTTGGGGAATACCAATATC | |
| AD-SlCAT1(568-1476)-F | gtaccagattacgctcatatgTTCGACGATATTGGTATTC | Y2H |
| AD-SlCAT1(568-1476)-R | atgcccacccgggtggaattcTATGCTTGGTCTCACATTAAGCCTAG | |
| AD-SlCAT1(1-441)-F | gtaccagattacgctcatatgATGGATCCATACAAGTATCG | Y2H |
| AD-SlCAT1(1-441)-R | atgcccacccgggtggaattcATCACGGATGAAGAAGACGG | |
| AD-SlCAT1(382-1476)-F | gtaccagattacgctcatatgATCACGGATGAAGAAGACGG | Y2H |
| AD-SlCAT1(382-1476)-R | atgcccacccgggtggaattcTATGCTTGGTCTCACATTAAGCCTAG | |
| AD-SlCAT1(1-240)-F | gtaccagattacgctcatatgATGGATCCATACAAGTATCG | Y2H |
| AD-SlCAT1(1-240)-R | atgcccacccgggtggaattcGTCATGGGTAACTTCAAAGA | |
| AD-SlCAT1(157-441)-F | gtaccagattacgctcatatgAATTTTGACAGGGAACGTGT | Y2H |
| AD-SlCAT1(157-441)-R | atgcccacccgggtggaattcATCACGGATGAAGAAGACGG | |
| AD-SlCAT1(1-231)-F | gtaccagattacgctcatatgATGGATCCATACAAGTATCG | Y2H |
| AD-SlCAT1(1-231)-R | atgcccacccgggtggaattcAACTTCAAAGAACCCTTTGGC | |
| AD-SlCAT1(1-216)-F | gtaccagattacgctcatatgATGGATCCATACAAGTATCG | Y2H |
| AD-SlCAT1(1-216)-R | atgcccacccgggtggaattcTTTGGCACTAGCACCTCGGGC | |
| YFP-C-SlCAT2-F | GAGAACACGGGGGACTCTAGAATGGATCCTTACAAGTACCGTC | BiFC |
| YFP-C-SlCAT2-R | GACAGTACTATCGATGGATCCTATGCTTGGTCTCACATTAAGC | BiFC |
| YFP-C-SlCAT1-F | GAGAACACGGGGGACTCTAGAATGGATCCCTCTAAGTATCGCC | BiFC |
| YFP-C-SlCAT1-R | GACAGTACTATCGATGGATCCCATTGTAGGCTTCACAGTGAGA | BiFC |
| YFP-N-p27-F | AGAACACGGGGGGACTCTAGAATGGAGGTCGTGTACAATTCAG | BiFC |
| YFP-N-p27-R | GACAGTACTATCGATGGATCCAATATAATAATTTGAGACTCTT | BiFC |
| 2-PROKII-p27-F | ACGGGGGACTCTAGAGGATCCATGGAGGTCGTGTACAATTCAG | subcellular localization, Co-IP |
| 2-PRROKII-p27-R | GCCCTTGCTCACCATGGTACCAATATAATAATTTGAGACTCTT | |
| SlCAT1-Mcs-dsRed-F | CTCTCTACAAGATCTATGGATCCCTCTAAGTATCGCC | subcellular localization |
| SlCAT1-Mcs-dsRed-R | CAGAATTCGAAGCTTTCATTGTAGGCTTCACAGTGAGA | |
| SlCAT2-Mcs-dsRed-F | CTCTCTACAAGATCTATGGATCCTTACAAGTACCGTC | subcellular localization |
| SlCAT2-Mcs-dsRed-R | CAGAATTCGAAGCTTTTATGCTTGGTCTCACATTAAGC | |
| pGD-SlCAT1-Flag-F | CGAATTCTGCAGTCGACGATGGATCCCTCTAAGTATCGCC | Co-IP |
| pGD-SlCAT1-Flag-R | TCTAGATCCGGTGGATCCTCACATTGTAGGCTTCACAGTGAGA | Co-IP |

| | | |
|---|---|---|
| pGD-SlCAT2-Flag-F | CGAATTCTGCAGTCGACGATGGATCCTTACAAGTACCGTC | Co-IP |
| pGD-SlCAT2-Flag-R | TCTAGATCCGGTGGATCCTCATATGCTTGGTCTCACATTAAGC | Co-IP |
| TRV-NbCAT1-F | AAGGTTACCGAATTCTCTAGACGAGGAGATCGACTACTTCC | VIGS |
| TRV-NbCAT1-R | CGTGAGCTCGGTACCGGATCCTTAAGCCTAGAAGCAAGCTT | |
| TRV-NbCAT2-F | AAGGTTACCGAATTCTCTAGATATCCCGAGTGGAAACTCT | VIGS |
| TRV-NbCAT2-R | CGTGAGCTCGGTACCGGATCCAAGCTGCATATAGTTTGGTCC | |
| NbCAT1-F | ATGGATCCATACAAGTATCGTC | RT-PCR detection |
| NbCAT1-R | TCATATGCTTGGTCTCACATTA | |
| NbCAT2-F | TATCCCGAGTGGAAACTCT | RT-PCR detection |
| NbCAT2-R | AAGCTGCATATAGTTTGGTCC | |
| ToCV p27-F | ATGGAGGTCGTGTACAATTCAG | RT-PCR detection |
| ToCV p27-R | TCAAATATAATAATTTGAGACT | |
| ToCV CP-F | ATGGAGAACAGTGCTGTTGCAA | RT-PCR detection |
| ToCV CP-R | TTAGCAACCAGTTATCGATGCA | |
| SlCAT1-F | ATGGATCCCTCTAAGTATCGCC | RT-PCR detection |
| SlCAT1-R | TCACATTGTAGGCTTCACAGTG | |
| 35S-F | GACGCACAATCCCACTATCC | RT-PCR detection |
| NOS-R | GATAATCATCGCAAGACCGG | |
| RT-qNbCAT1-F | GTTCTACACCAGAGAGGGAAAC | qRT-PCR |
| RT-qNbCAT1-R | GACCATGTCAGGGAACTTCAT | |
| RT-qNbCAT2-F | TTTGATCCTCTTGATGTAAC | qRT-PCR |
| RT-qNbCAT2-R | TCAGCATACGCGAATATCC | |
| RT-qToCVCP-F | CCGTTAGATGATGGCGTAGATG | qRT-PCR |
| RT-qToCVCP-R | GCCCAACTGGAAGATCGATTAG | |
| RT-qPVXCP-F | CAGGGTCAACTACCTCAACTAC | qRT-PCR |
| RT-qPVXCP-R | GGCACGAGCTGTACTAAAGAA | |
| p27-QATG-F | GAGGTCGTGTACAATTCAGACG | qRT-PCR |
| p27-QTGA-R | AATATAATAATTTGAGACTCTT | |
| RT-qPVX97-F | CTGCTGCCTTTGTGAAGATTAC | qRT-PCR |
| RT-qPVX97-R | GTTGTTCCAGTGATACGACCT | |
| EF-1α-F | TGAGATGCACCACGAAGCTC | qRT-PCR |
| EF-1α-R | CCAACATTGTCACCAGGAAGTG | |
